# Splenectomy Linking Guillain-Barré Syndrome, Autoimmune Haemolysis, and Epstein-Barr Virus Viraemia: Unveiling a Hidden Diagnosis of Angioimmunoblastic T-cell Lymphoma

**DOI:** 10.7759/cureus.95571

**Published:** 2025-10-28

**Authors:** Pranav Santhosh Kumar, Thomas Quarrell, Livia Raso-Barnett, Jonathan Knight, Tom Bull

**Affiliations:** 1 Haematology, West Suffolk Hospital, Bury St Edmunds, GBR; 2 Internal Medicine, Norfolk and Norwich University Hospital, Norwich, GBR; 3 Pathology, Cambridge University Hospitals, Cambridge, GBR

**Keywords:** aitl, angioimmunoblastic t-cell lymphoma, autoimmune hemolytic anemia (aiha), ebstein barr virus, guillan-barré syndrome (gbs), nodal t-follicular helper cell lymphoma, splenic biopsy

## Abstract

A 64-year-old man presented to the emergency department of a district general hospital with a widespread rash. He received antihistamine treatment and was discharged. He subsequently returned with peripheral paraesthesia and severe back pain. Neurological assessment showed pronounced bilateral ascending lower limb motor weakness, and further review found diplopia and dysphagia. A diagnosis of Guillain-Barré syndrome (GBS) with the Miller Fisher variant was established, and treatment with high-dose intravenous immunoglobulin (IVIG) was initiated. The patient was transferred to a tertiary centre for nerve conduction studies.

During repatriation, the patient developed severe hyponatraemia, and paired serum and urine osmolality testing indicated syndrome of inappropriate antidiuretic hormone secretion (SIADH). A staging CT discovered numerous borderline enlarged lymph nodes, seemingly consistent with a viral screen, which identified an Epstein-Barr virus (EBV) viraemia. A rapid drop in the patient’s haemoglobin alongside a positive direct antibody test prompted a further diagnosis of autoimmune haemolytic anaemia (AIHA). High-dose steroids and rituximab both failed to abate the haemolysis, necessitating an ITU admission for stabilisation and emergency splenectomy.

Histopathological analysis of the spleen was consistent with angioimmunoblastic T-cell lymphoma (AITL), providing a unifying explanation for the patient’s diverse autoimmune manifestations. The patient was initiated on R-CHOP (rituximab, cyclophosphamide, doxorubicin, vincristine, and prednisone) chemotherapy, and he achieved a complete metabolic response on interim PET-CT, and remains in remission at nine months of follow-up. This presentation of AITL with concurrent GBS and AIHA highlights the potential for this disease to present primarily with features of immune dysregulation.

## Introduction

Nodal T-follicular helper cell lymphoma, angioimmunoblastic type, commonly referred to as angioimmunoblastic T-cell lymphoma (AITL), accounts for 20% of peripheral T-cell lymphoma cases [1] with an incidence of 0.05 in every 100,000 new patients in the US [2]. Clinical features on presentation can include classical B symptoms (fever, weight loss, and night sweats), lymphadenopathy, hepatosplenomegaly, and autoimmune mimicry [3]. We describe the case of a previously healthy 64-year-old male who was diagnosed and treated for AITL. 

Parts of this work were previously presented as a poster at the British Society for Haematology 65th Annual Scientific Meeting on April 28, 2025.

## Case presentation

A 64-year-old male presented to the emergency department with a widespread macular rash that developed after a suspected weever fish sting. He had a past medical history of migraines for which he took propranolol as prophylaxis. He received antihistamines and a tetanus booster before being discharged. Six days later, he returned with peripheral paraesthesia and severe back pain. 

First impression: identifying Guillain-Barré syndrome

Admission blood tests were unremarkable - haemoglobin (Hb): 137 g/L, white blood cell count (WCC): 9.2 × 10⁹/L, C-reactive protein (CRP): 7 mg/L, sodium: 140 mmol/L, potassium: 4.3 mmol/L, and calcium: 2.13 mmol/L. Baseline ECG showed normal sinus rhythm with normal axis, T-wave inversion in lead III, PR interval of 135ms, and QTc 431 ms. The patient developed motor impairment, which primarily affected the proximal lower limbs. Initial neurological examination showed reduced power bilaterally in hip flexion, knee flexion and extension, and right ankle plantarflexion, with Medical Research Council (MRC) scores of 3/5. Over the next few days, the patient developed ophthalmoplegia and bulbar dysfunction.

A clinical diagnosis of Miller Fisher syndrome was established, and the weever fish sting was ultimately considered coincidental. This diagnosis was reaffirmed by lumbar puncture and nerve conduction studies (Table 1). The patient was treated with a five-day course of 2 g/kg intravenous immunoglobulin, which was interrupted by three days of 1g IV methylprednisolone while the diagnosis of transverse myelitis was being evaluated. During treatment, his bulbar and ocular function improved, and his forced vital capacity remained stable. 

**Table 1:** Summary of neurological investigations CT: computed tomography; MRI: magnetic resonance imaging; GBS: Guillain-Barré syndrome; WCC: white blood cell count

Investigation	Result
CT head	Normal
MRI spine	Mild cauda equina ventral thickening suggestive of GBS
Nerve conduction study	Patchy demyelination, compatible with acute inflammatory demyelinating pathology. No strong evidence for the atypical variant
Lumbar puncture	Elevation in protein without elevation in WCC; albuminocytologic dissociation consistent with GBS
Borrelia serology	Negative
Autoimmune screen	Negative

Prolonged hyponatraemia: investigating syndrome of inappropriate antidiuretic hormone secretion (SIADH)

Following this, the patient developed pyrexia of unknown origin (PUO) and severe hyponatraemia. Clinical assessment and paired serum and urine osmolality and sodium results indicated euvolemic hyponatraemia leading to a diagnosis of syndrome of inappropriate antidiuretic hormone secretion (SIADH) (Table 2). The hyponatraemia was refractory to fluid restriction and required demeclocycline before normalisation of sodium levels. 

**Table 2:** Summary of investigations for hyponatraemia TSH: thyroid-stimulating hormone

Investigations	Reference range	Results
Serum sodium (mmol/L)	133-146	119
Serum osmolarity (mOsm/kg)	275-295	257
Urine osmolarity (mOsm/kg)	50-1200	557
Urine sodium (mmol/L)	40-220	46
TSH (mU/L)	0.27-4.2	1.65
Random cortisol (nmol/L)	140-690	423

**Figure 1:**
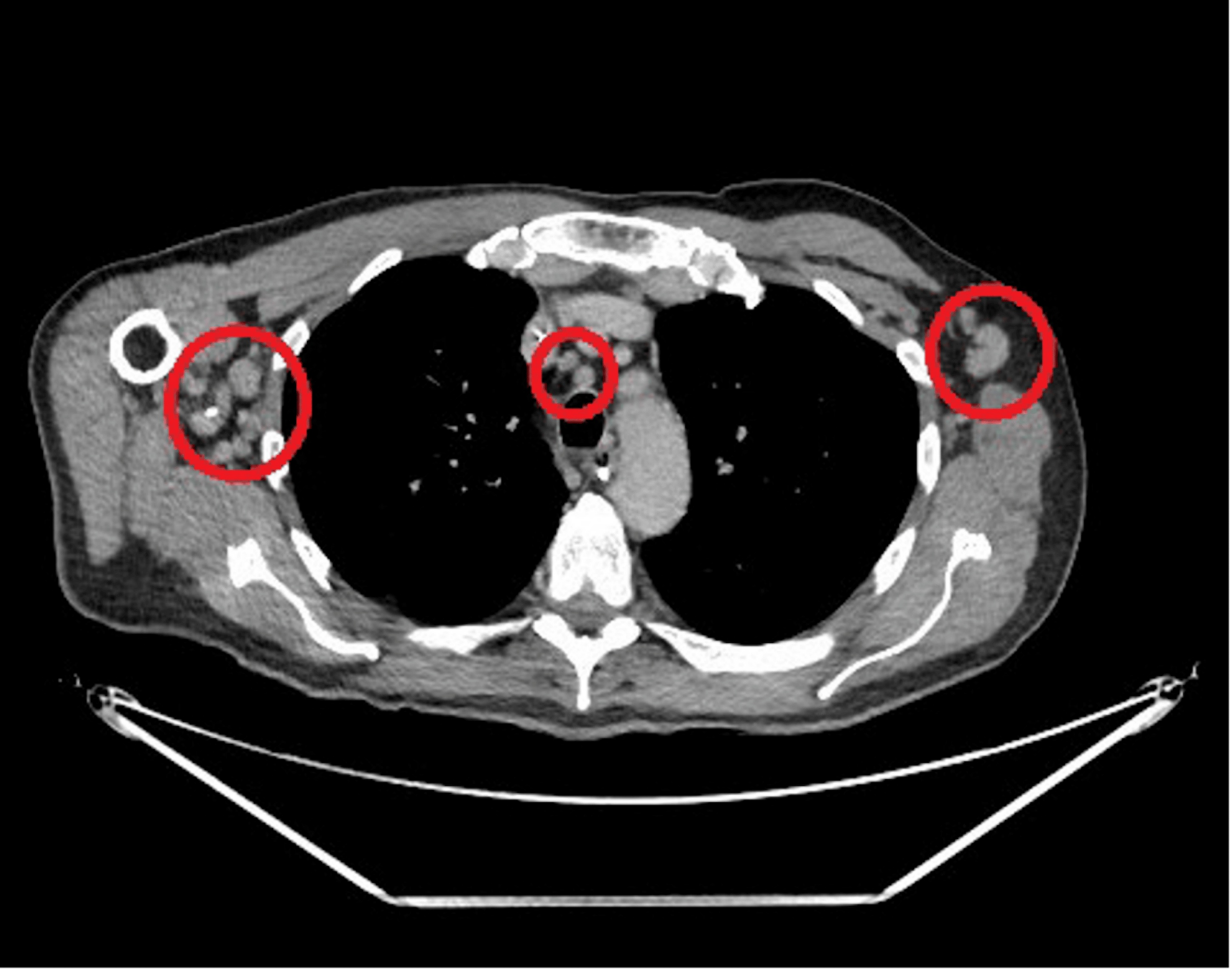
Axillary and pre-tracheal lymphadenopathy indicated on CT-scan The largest nodes seen are 10 mm in diameter CT: computed tomography

The prolonged SIADH in the context of PUO prompted a CT scan of the chest, abdomen, and pelvis to rule out occult malignancy as a cause. This revealed numerous borderline enlarged lymph nodes seen in the pre-tracheal, axillary and inguinal spaces (Figure 1), which were not amenable to lymph node biopsy due to size.

Expanding the clinical puzzle: EBV viraemia, erythema multiforme, and autoimmune haemolytic anaemia

Given the persistent PUO, a viral screen was performed to investigate the cause of the fever and lymphadenopathy. This revealed that the patient had an EBV viraemia with serology positive for EBV IgM and IgG and a viral load of 5.8 x 10³ copies/mL, indicating a potentially reactive explanation for the borderline enlarged lymph nodes. Shortly after the normalisation of his sodium, the patient developed widespread papulovesicular, target-like lesions on his trunk, upper arms and back with palm and sole involvement. A dermatology review made a diagnosis of erythema multiforme, and treatment with high-dose prednisolone was recommended. This too was thought to be related to ongoing EBV viraemia.

A rapid decline in the patient’s haemoglobin level, in the absence of any apparent bleeding, subsequently prompted evaluation for autoimmune haemolytic anaemia (AIHA). Tests for AIHA confirmed the diagnosis, with pertinent findings including a positive direct antibody test, raised reticulocyte count, and an unconjugated bilirubinaemia (Table 3).

**Table 3:** Summary of investigations for AIHA AIHA: autoimmune haemolytic anaemia; Hb: haemoglobin; LDH: lactate dehydrogenase

Investigations	Reference range	Results
Hb (g/L)	130-180	76
Reticulocyte count (10*9/L)	30-100	286.9
Blood film		Occasional polychromasia. Occasional toxic granulations in neutrophils
LDH (U/L)	225	421
Unconjugated bilirubin (µmol/L)	0-20	37
Direct antibody test		AHG 4+, IgG 4+, IgA 0+, IgM 2+, C3c 0, C3d 3+, C4 0, control 0

Bone marrow aspirate and trephine were performed to exclude a lymphoproliferative disorder as a secondary cause of AIHA. This showed hypercellular marrow, evidencing expanded erythropoiesis and ongoing haemolysis. Mixed lymphoid aggregates were seen with inconclusive PCR for T-cell and B-cell clonality. The haemolysis was refractory to high-dose steroids (intravenous methylprednisolone 1 mg/kg), intravenous immunoglobulins (single 1g/kg dose) and rituximab (375 mg/m^2^ weekly for four weeks), culminating in an emergency splenectomy. Following the splenectomy, there was a steady improvement in all haemolytic markers, and the patient became transfusion-independent approximately 10 days after the surgery.

Unifying a myriad diagnoses: insights from the splenic biopsy

**Figure 2:**
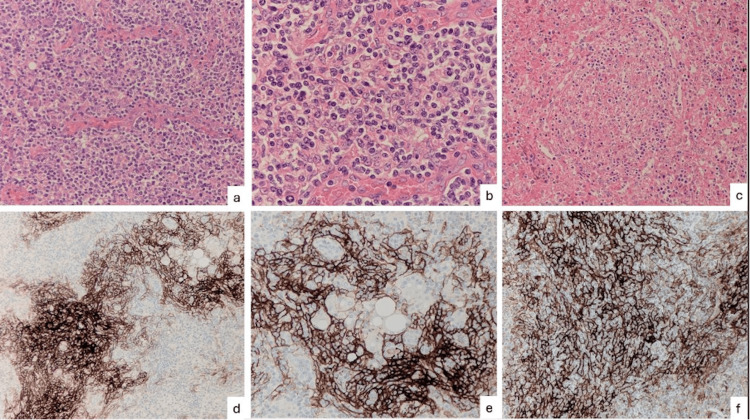
Lymph node and spleen histology - architecture and FDC network (a) Lymph node HE-stained (x10). (b) Lymph node HE-stained (x20). (c) Spleen HE-stained (x10). (d) Lymph node stained for CD21 (x10). (e) Lymph node stained for CD21 (x20). (f) Spleen stained for CD21 (x20) FDC: follicular dendritic cells

**Figure 3:**
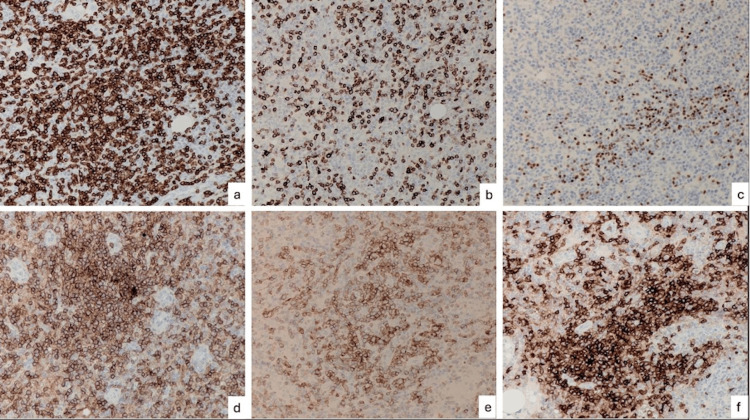
Lymph node histology 2 (a) Stained for CD4 (x20). (b) Stained for CD7 (x20). (c) Stained for PAX5 (x20). (d) Stained for CD4 (x20). (e) Stained for PD1 (x20). (f) Stained for CD10 (x20)

**Figure 4:**
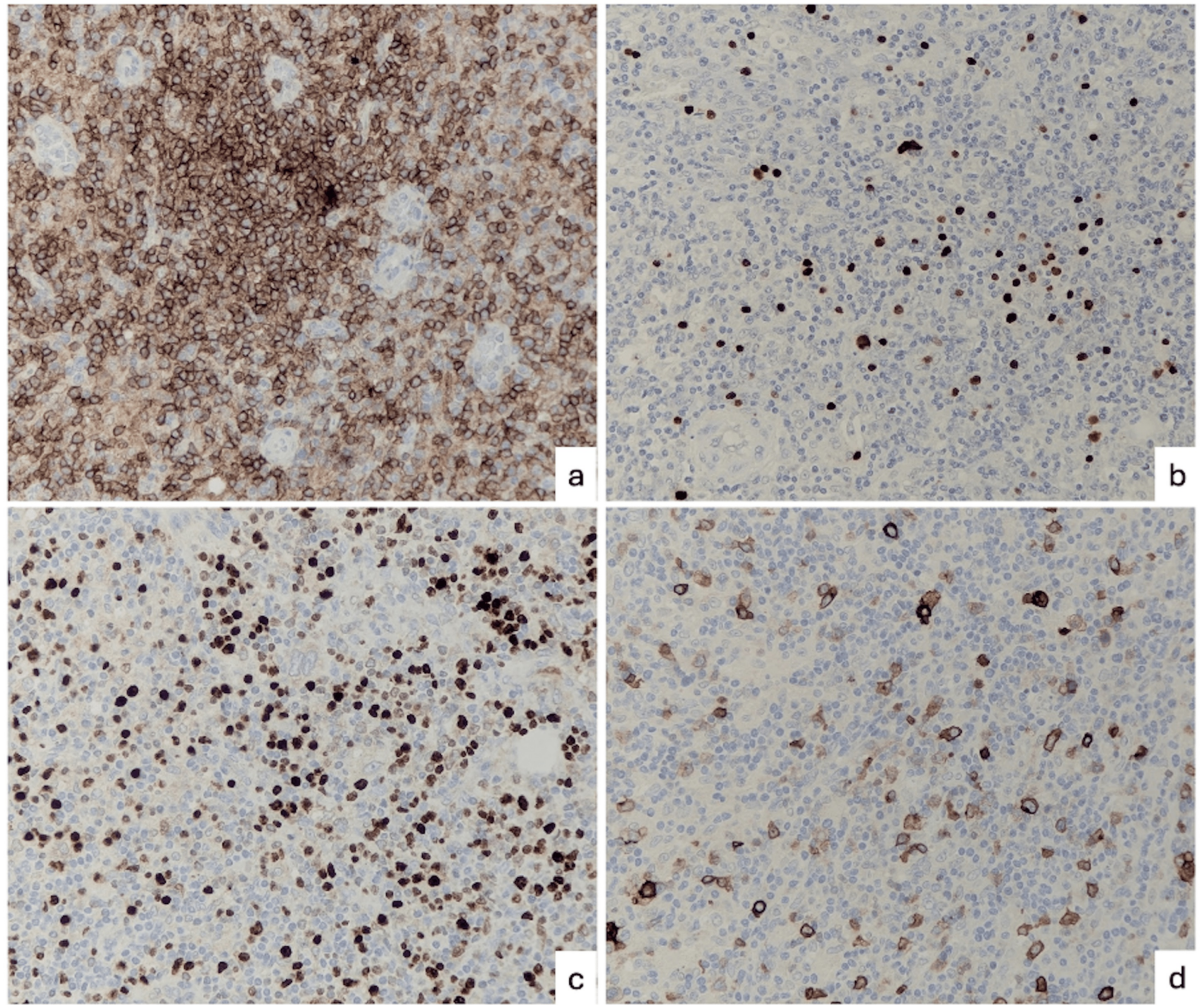
Lymph node histology 3 (a) Stained for CD4 (x20). (b) EBER-ISH (x20). (c) Stained for Ki67 (x20). (d) Stained for CD30 (x20)

Laparoscopic splenectomy was performed by an open Hassan approach via infraumbilical incision. Macroscopically, the spleen was enlarged with a bulky, dense hilum and dense omental adhesions overlying it. Microscopic examination showed the architecture of the spleen as well as the hilar lymph node to be effaced. Associated with prominent high endothelial venules (Figures 2a-2c) were prominent expanded FDC-meshworks (Figures 2d-2f) and a population of medium-sized atypical CD4+/PD1+ T-follicular helper cells (Figures 3d-3e) with preserved CD2, CD3 (Figure 3a) and CD5 but reduced CD7 (Figure 3b) expression as well as increased CD10 staining (Figure 3f). PAX5 (Figure 3c), EBER-ISH (Figure 4b) and CD30 (Figure 4d) highlighted scattered immunoblasts. The proliferation index within the neoplastic T-cell population was 20-30% (Figure 4c). Haem-Onc NGS sequencing panel showed pathogenic mutations in the TET2 and RHOa genes.

The histological changes described in conjunction with the evidence of clonal T-cell receptor rearrangement and pathognomonic mutations were consistent with the diagnosis of AITL. The patient received six cycles of standard-dose R-CHOP (rituximab, cyclophosphamide, doxorubicin, vincristine, and prednisolone) chemotherapy, achieving a complete metabolic response on interim PET-CT after two cycles. He remains in complete remission nine months into follow-up.

## Discussion

The autoimmune masquerade: angioimmunoblastic T-cell lymphoma

This report discussed a case of AITL presenting initially with multiple features of immune dysregulation, namely GBS, erythema multiforme and AIHA. The patient later developed the more classical B-symptoms with fever, and the diagnosis only became evident after the splenic biopsy. An analysis of the Peripheral T-cell Lymphoma Project has shown that lymphadenopathy was present in 76% of patients, along with rash in 21% of patients and AIHA in 13% of patients [2].

**Figure 5:**
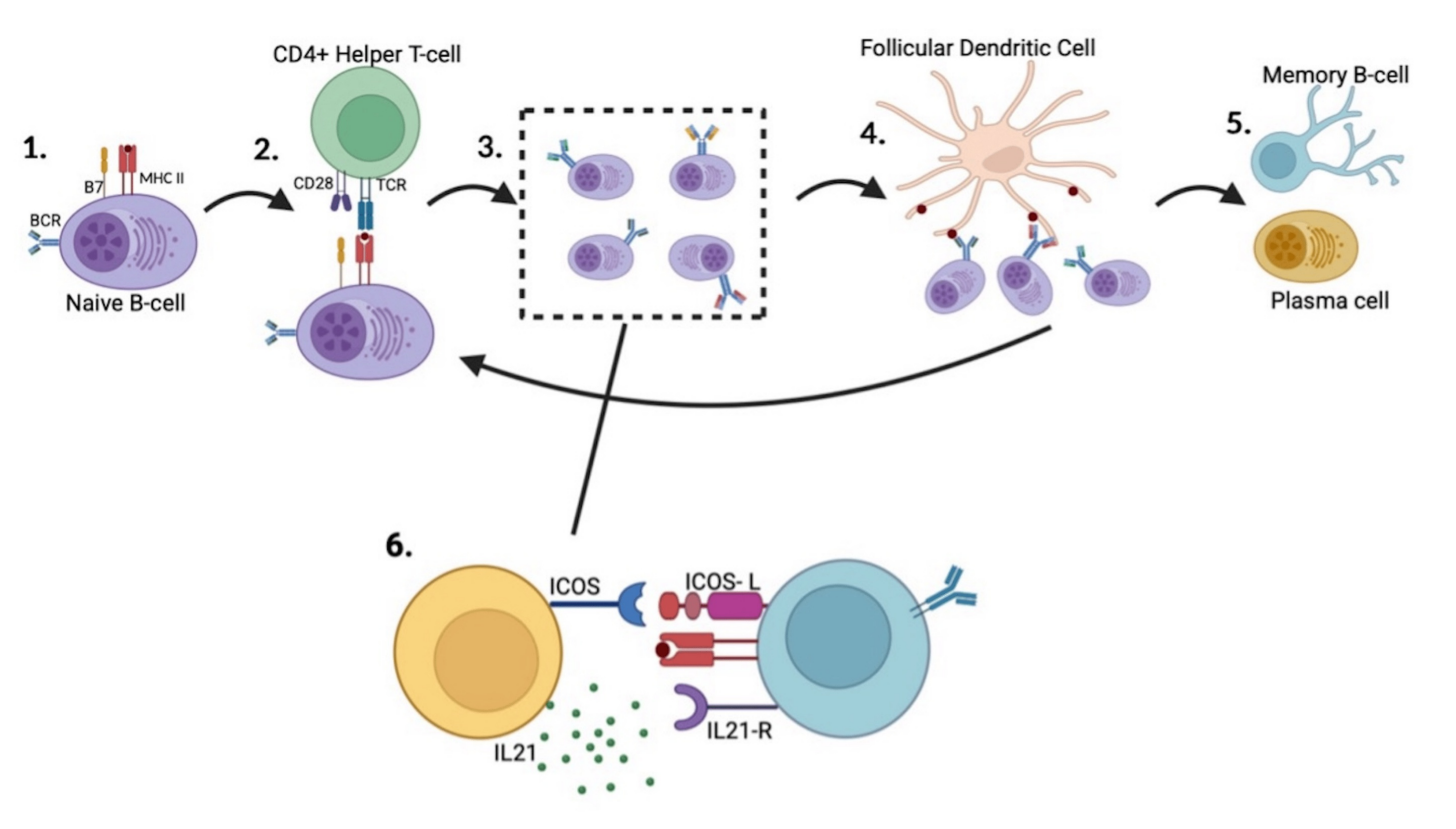
Affinity maturation in germinal centres and the role of the T-follicular helper cell 1. Naive B-cell enters the germinal centre expressing an antigen on an MHC II. 2. TCR/MHC-II interaction, along with co-stimulation by CD28-B7, results in B-cell activation. 3. The processes of somatic recombination and somatic hypermutation produce antibody diversity in the B-cell population. 4. Follicular dendritic cells present antigens to activated B-cell BCRs, and the process of affinity maturation selects for higher-affinity interactions in an iterative process. 5. Clonal expansion of plasma cells with high-affinity antibodies and memory cells. 6. T-follicular helper cells aid in the formation of germinal centres with ICOS, IL-21, and CD40 signals promoting B-cell proliferation [5] Image created using BioRender.com. Image credits: Pranav Santhosh Kumar

The cell of origin in AITL is the T-follicular helper cell, which is involved in the process of affinity maturation and plasma cell development in a cell-mediated immune response (Figure 5) [4].

Dysregulation of this process is thought to be due to IL-4 overexpression and an ICOS positive feedback loop, which further reinforces the germinal centre microenvironment for polyclonal B-cell expansion. Autoantibody production is subsequently thought to result in the various immunological manifestations of AITL [5]. This case is notable due to the fulminant and multifaceted complicating features, which obscured the path to diagnosis. The initial diagnosis of AITL was not established from the bone marrow biopsy, despite bone marrow involvement being observed in approximately 70% of AITL cases [6], but was instead made following examination of a secondary lymphoid organ. Additionally, investigation by lymph-node biopsy was not possible due to their small size. Other cases with a similar constellation of immunological manifestations are detailed in Table 4. A literature search was performed on PubMed, eliciting case reports by using the keywords "angioimmunoblastic T-cell lymphoma, autoimmune haemolytic anaemia, and Guillain-Barré syndrome."

**Table 4:** Summary of published case reports with similar immunological manifestations of AITL AITL: angioimmunoblastic T-cell lymphoma; LL: lower limb; PUO: pyrexia of unknown origin; Hb: haemoglobin; WCC: white blood cell count; CSF: cerebrospinal fluid; NCS: nerve conduction study; GBS: Guillain-Barré syndrome; CTCAP: computed tomography scan of the chest, abdomen, and pelvis; AIHA: autoimmune haemolytic anaemia; LDH: lactate dehydrogenase; DAT: direct antiglobulin test; IgG: immunoglobulin G; FISH: fluorescence in situ hybridisation test; EBV: Epstein-Barr virus

Study	Patient age, years	Presentation and examination findings	Lab findings	Histology/immunophenotype
Howell et al. (2022) [7]	80s	Fatigue, breathlessness, proximal LL weakness, frequent falls	Hb: 108, WCC: 3.5, reticulocytes: 16%, platelets: 90, bilirubin: 46, CSF: albuminocytological dissociation, NCS: severely slowed velocities, GBS diagnosed	Lymph node: heavy involvement of polymorphous neoplastic cells; vascular proliferation seen; CD3, 4, 5, 57, BCL6, and PD-1 + cells seen; FISH negative for EBV; AITL diagnosed
Pathak et al. (2018) [8]	71	Breathlessness, generalised weakness, LL paraesthesia	Hb: 56, WCC: 4.8, reticulocytes: 2.12%, CTCAP: para-aortic lymphadenopathy, CSF: albuminocytological dissociation, GBS diagnosed	Bone marrow: hypercellular marrow, nil evidence of malignancy; cervical lymph node: CD3, 4, 10, BLC6 + with EBV + B-cells; AITL diagnosed
Talreja et al. (2025) [9]	48	Weakness, jaundice, breathlessness, PUO, hepatosplenomegaly	Hb: 51; WCC: 14.5; platelets: 19; AIHA diagnosed; CTCAP: axillary, mediastinal, mesenteric, and inguinal lymphadenopathy; serum chain electrophoresis: polyclonal gammaglobinaemia	Lymph node: effaced architecture; prominent high endothelial venules; CD3, 20, 79a + CD23, 21 + in dendritic meshwork; AITL diagnosed
Shida et al. (2011) [10]	44	Generalised lymphadenopathy, fever, anaemia, jaundice	Hb: 54; WCC: 1.68; reticulocytes: 73%; unconjugated bilirubinaemia; LDH: 520; DAT: IgG and C3d ++ HIV Ab +, but HIV RNA -ive; AIHA diagnosed	Axillary lymph node: effaced architecture; arborising vessels, CD2, 3,5, 7, 10 + cells, FDC with CD21 + occasional EBV + plasma cells; AITL diagnosed

These reports describe patients initially presenting with GBS or AIHA who were subsequently diagnosed with AITL; however, the simultaneous occurrence of these conditions has not been previously documented. Interestingly, in this case, the presence of an EBV viraemia could also have contributed to some of the presentations described, such as SIADH, erythema multiforme and even GBS. Reports of EBV infection causing antibody cross-reaction and subsequent peripheral demyelination [11] and persistent erythema multiforme [12] have been documented. Equally, EBV infection of polyclonal B cells was found in 85-95% of AITL biopsies, with the lymphoproliferative effects of the virus postulated to contribute to the overall disease process [13]. It is therefore important to determine whether the EBV viraemia is a feature of the case or serves as a primary driving factor.

## Conclusions

This report highlights the importance of a thorough and iterative diagnostic approach in complex autoimmune cases, providing valuable insights for clinicians. AITL may first present with immune-mediated complications, including GBS and AIHA. Recognition of this constellation, particularly in the presence of EBV viraemia, should prompt early consideration of AITL and tissue diagnosis. While this single case report cannot establish causality, the temporal sequence and histopathological confirmation support AITL as the unifying process. Earlier diagnosis can result in improved trial recruitment and hopefully result in better treatment outcomes for AITL.
